# Brainhack: a collaborative workshop for the open neuroscience community

**DOI:** 10.1186/s13742-016-0121-x

**Published:** 2016-03-31

**Authors:** R. Cameron Craddock, Daniel S. Margulies, Pierre Bellec, B. Nolan Nichols, Sarael Alcauter, Fernando A. Barrios, Yves Burnod, Christopher J. Cannistraci, Julien Cohen-Adad, Benjamin De Leener, Sebastien Dery, Jonathan Downar, Katharine Dunlop, Alexandre R. Franco, Caroline Seligman Froehlich, Andrew J. Gerber, Satrajit S. Ghosh, Thomas J. Grabowski, Sean Hill, Anibal Sólon Heinsfeld, R. Matthew Hutchison, Prantik Kundu, Angela R. Laird, Sook-Lei Liew, Daniel J. Lurie, Donald G. McLaren, Felipe Meneguzzi, Maarten Mennes, Salma Mesmoudi, David O’Connor, Erick H. Pasaye, Scott Peltier, Jean-Baptiste Poline, Gautam Prasad, Ramon Fraga Pereira, Pierre-Olivier Quirion, Ariel Rokem, Ziad S. Saad, Yonggang Shi, Stephen C. Strother, Roberto Toro, Lucina Q. Uddin, John D. Van Horn, John W. Van Meter, Robert C. Welsh, Ting Xu

**Affiliations:** The Neuro Bureau, Leipzig, 04317 Germany; Computational Neuroimaging Lab, Center for Biomedical Imaging and Neuromodulation, Nathan S. Kline Institute for Psychiatric Research, Orangeburg, New York, 10962 USA; Center for the Developing Brain, Child Mind Institute, New York, New York, 10022 USA; Max Planck Research Group for Neuroanatomy & Connectivity, Max Planck Institute for Human Cognitive and Brain Sciences, Leipzig, 04103 Germany; Département d’Informatique et de Recherche Opérationnelle, Université de Montréal, Montréal, Québec H3W 1W5, Canada; Functional Neuroimaging Unit, Centre de Recherche de l’Institut Universitaire de Gériatrie de Montréal, Montréal, Québec H3W 1W5, Canada; Center for Health Sciences, SRI International, Menlo Park, California, 94025 USA; Department of Psychiatry and Behavioral Sciences, Stanford University, Stanford, California, 94305 USA; Instituto De Neurobiología, Universidad Nacional Autónoma de México, Querétaro, 76203 México; Laboratoire d’Imagerie Biomédicale, Sorbonne Universités, UPMC Université Paris 06, Paris, 75005 France; Institut des Systèmes Complexes de Paris-Île-de-France, Paris, 75013 France; Translational and Molecular Imaging Institute, Icahn School of Medicine at Mount Sinai, New York, New York, 10029 USA; Institute of Biomedical Engineering, Ecole Polytechnique de Montréal, Montréal, Québec H3T 1J4, Canada; McConnell Brain Imaging Center, Montreal Neurological Institute, Montreal, Quebec H3A 2B4, Canada; MRI-Guided rTMS Clinic, University Health Network, Toronto, Ontario M5T 2S8, Canada; Department of Psychiatry, University Health Network, University of Toronto, Toronto, Ontario M5T 2S8, Canada; Institute of Medical Sciences, University of Toronto, Toronto, Ontario M5S 1A8, Canada; Faculdade de Engenharia, PUCRS, Porto Alegre, 90619 Brazil; Instituto do Cérebro do Rio Grande do Sul, PUCRS, Porto Alegre, 90610 Brazil; Faculdade de Medicina, PUCRS, Porto Alegre, 90619 Brazil; New York State Psychiatric Institute, New York, New York, 10032 USA; Division of Child and Adolescent Psychiatry, Department of Psychiatry, Columbia University, New York, New York, 10032 USA; McGovern Institute for Brain Research, Massachusetts Institute of Technology, Cambridge, Massachusetts, 02139 USA; Department of Otology and Laryngology, Harvard Medical School, Boston, Massachusetts, 02115 USA; Department of Radiology, University of Washington, Seattle, Washington, 98105 USA; Department of Neurology, University of Washington, Seattle, Washington, 98105 USA; International Neuroinformatics Coordinating Facility, Stockholm, 171 77 Sweden; Karolinska Institutet, Stockholm, 171 77 Sweden; Faculdade de Informática, PUCRS, Porto Alegre, 90619 Brazil; Center for Brain Science, Harvard University, Cambridge, Massachusetts, 02138 USA; Department of Physics, Florida International University, Miami, Florida, 33199 USA; Chan Division of Occupational Science and Occupational Therapy, Division of Physical Therapy and Biokinesiology, Department of Neurology, University of Southern California, Los Angeles, California, 90033 USA; USC Mark and Mary Stevens Neuroimaging and Informatics Institute, University of Southern California, Los Angeles, Canada, 90033 USA; Department of Psychology,, University of California, Berkeley, California, 94720 USA; Biospective, Inc., Montréal,, Québec H4P 1K6, Canada; Department of Neurology, Massachusetts General Hospital, Boston, Massachusetts, 02114, USA; Radboud University Nijmegen, Donders Institute for Brain, Cognition and Behaviour, Centre for Cognitive Neuroimaging, Nijmegen, 6525 EN The Netherlands; Sorbonne Universités, Paris-1 Université, Equipement d’Excellence MATRICE, Paris, 75005, France; Functional MRI Laboratory, University of Michigan, Ann Arbor, Michigan, 48109 USA; Helen Wills Neuroscience Institute, University of California, Berkeley, California, 94720 USA; Henry H. Wheeler Jr. Brain Imaging Center, University of California, Berkeley, California, 94709 USA; Laboratory of Neuro Imaging, Stevens Neuroimaging and Informatics Institute, Keck School of Medicine of University of Southern California, Los Angeles, California, 90033 USA; The University of Washington eScience Institute, Seattle, Washington, 98195 USA; Scientific and Statistical Computing Core, National Institute of Mental Health, Bethesda, Maryland, 20892 USA; Rotman Research Institute, Baycrest Hospital, Toronto, Ontario M6A 2E1, Canada; Department of Medical Biophysics, University of Toronto, Toronto, Ontario M5G 1L7, Canada; Human Genetics and Cognitive Functions Unit, Institut Pasteur, Paris, 75015 France; Unité Mixte de Recherche 3571, Genes, Synapses and Cognition, Centre National de la Recherche Scientifique, Institut Pasteur, Paris, 75015 France; Department of Psychology, University of Miami, Coral Gables, Florida, 33124 USA; Neuroscience Program, University of Miami Miller School of Medicine, Miami, Florida, 33136 USA; Center for Functional and Molecular Imaging, Georgetown University Medical Center, Washington,, 20007 DC USA; Department of Psychiatry, University of Michigan, Ann Arbor, Michigan, 48109 USA; Department of Radiology,, University of Michigan, Ann Arbor, Michigan, 48109 USA

**Keywords:** Hackathon, Unconference, Open science, Neuroscience, Data sharing, Collaboration, Networking

## Abstract

Brainhack events offer a novel workshop format with participant-generated content that caters to the rapidly growing open neuroscience community. Including components from hackathons and unconferences, as well as parallel educational sessions, Brainhack fosters novel collaborations around the interests of its attendees. Here we provide an overview of its structure, past events, and example projects. Additionally, we outline current innovations such as regional events and post-conference publications. Through introducing Brainhack to the wider neuroscience community, we hope to provide a unique conference format that promotes the features of collaborative, open science.

## Introducing Brainhack

Open science promotes collaboration through the transparent dissemination of ideas, tools, and data, with the goal of accelerating the pace of discovery. Although scientific conferences and workshops seem like a natural medium for brain researchers to meet and exchange ideas, in practice these events, often structured around the lecturer–audience paradigm, do not always provide sufficient flexibility or free time to fully exploit their potential. Borne from the technology sector, ‘unconferences’ and ‘hackathons’ are alternative meeting formats that emphasize the full participation of all attendees. Rather than having a prearranged program, the content presented at unconferences is dynamically determined by attendees, while hackathons feature unstructured time during which teams of participants collaborate intensively on various projects. These meeting formats have enabled rapid advances in computing technologies since the late 1990s, but they have yet to be widely adopted in academic research.

**Fig. 1:**
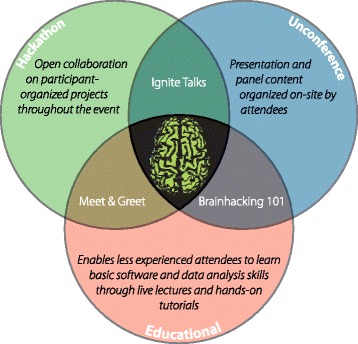
Brainhack [1] is composed of various organizational features from ‘unconferences’ and ‘hackathons,’ and includes a variety of scheduling components to encourage collaboration and introduction to open science methods

Now in their fourth year, international and regional Brainhack events bring together brain enthusiasts from a variety of backgrounds to build relationships, learn from one another, and collaborate on projects related to the neurosciences. Unlike traditional hackathons that tend to focus on computer programming, projects at Brainhacks can be completed using a much broader array of methods. Time is set aside for periodic unconference sessions whose content is determined on-site by the participants. The unconference sessions can feature different styles of presentations, including but not limited to: updates on ongoing projects, ideas that could seed future collaborations, panel discussions, or tutorials. In consideration of the ever expanding interest in the tools of open science, Brainhack has developed an educational component that runs in parallel with the hacking sessions in order to introduce the basic tools of open collaboration. This combined model encourages active participation and interaction between attendees, while also maximizing the topical relevance of the more formally presented content (see Fig. 1).

There is no ideal background, skill set, or experience level required for Brainhack attendees. Fully translating neuroscience data to knowledge requires expertise that spans biology, computer science, engineering, informatics, mathematics, neuroanatomy, philosophy, physics, psychiatry, psychology, statistics, art, and many others. The goal of a Brainhack event is to facilitate the cross-pollination of ideas and knowledge across these various disciplines and communities to accelerate the development of a richer understanding of the brain. In addition to sharing data and tools, attendees can contribute in a variety of ways. Philosophical debates about the meanings of cognition, coordinated efforts to manually segment brain images from different species, curating neuroscience literature, or helping others to understand the subtleties of diagnosing a developmental disorder are all examples of valuable contributions that have emerged at Brainhacks in the past (for further examples, see Table 1).

**Table 1:** Selected examples of Brainhack projects

∙ A child psychiatrist and a 3D video artist initiated a collaboration at the 2012 Brainhack to develop a movie to be shown to participants during resting-state fMRI scans to reduce head motion in hyperkinetic populations [5, 6].
∙ The ABIDE Preprocessing Initiative [7] is an ongoing project started at the 2012 Brainhack to share preprocessed versions of the Autism Brain Imaging Data Exchange (ABIDE) dataset [8, 9]. This project is sharing functional data that have been processed using the Connectome Computation System (CCS) [10, 11], the Configurable Pipeline for the Analysis of Connectomes (C-PAC) [12, 13], the Data Preprocessing Assistant for Resting State fMRI (DPARSF) [14, 15], and the Neuro Imaging Analysis Kit (NIAK) [16, 17], as well as cortical thickness measures extracted from structural data using FreeSurfer [18, 19], CIVET [20, 21], and Advanced Normalization Tools (ANTS) [22, 23].
∙ A collaboration started at the 2012 Brainhack performed an analysis to identify differences in cortical thickness and structural covariance between individuals with autism spectrum disorder and neurotypical controls [24].
∙ A project team at Brainhack 2013 amassed a dataset of 14,781 structural MRI scans to estimate the distribution of brain sizes across individuals for optimizing scan acquisition parameters [25].
∙ The development team of LORIS, an open source database system for neuroimaging and phenotypic data, have repeatedly used Brainhack as an opportunity to meet and collaborate on new features [26].
∙ An early version of the Daydreaming app [27], an Android application for real-time assessment of users’ mind-wandering, was developed at Brainhack 2013.
∙ The Clubs of Science [28] project, founded at Brainhack MTL 2015, has built a web-based visualization of the social web underlying neuroimaging research.
∙ The linkRbrain [29] tool for integrating and querying neuroimaging data with activation peaks from the literature and gene expression data was partially developed and first tested at Brainhack 2013 in Paris [30].

Further projects can be found at https://www.brainhack.org [1]

### Hackathons based on collaboration, not competition

The hackathon format gained prominence in the technology sector by providing a meeting model that targets specific project goals during intense time-limited collaborations. The competitive aspect of the traditional hackathon, while catalyzing rapid advances toward specific technology ends, is contrary to the founding principle of Brainhack, which is to encourage open, cross-institutional, and inter-disciplinary collaboration. Rather than subdividing attendees into competitive factions, Brainhack attendees are encouraged to work together in collaborative teams to solve problems of their choosing. In this way, rather than obtaining many solutions to a single problem, we aim to produce various solutions to many problems. Most importantly, we encourage the building of new relationships that continue to be productive beyond the end of the event.

### Brainhack projects

Rather than focusing on a specific problem or toolset, which is common in traditional hackathons, attendees are encouraged to generate their own project ideas around which they can self-assemble into teams. As a consequence, some projects may receive more limited interest, whereas one might attract the majority of attendees. In this way, the projects developed at Brainhacks are elected by participation, rather than being pre-specified by the event organizers. This model is more conducive to collaboration than the alternative of organizing a hackathon around a challenge or competition. There are many different models between these two extremes; we are currently working on building thematic Brainhack events that bring researchers together to focus on specific questions of neuroscientific interest.

To date, projects completed at Brainhack events have included students interacting with more experienced researchers to learn about a new data modality or analysis method, inter-disciplinary collaborations to improve data collection, the development or optimization of data analysis tools, and testing hypotheses about brain structure using openly shared data. See Table 1 for selected examples of projects that have previously been initiated at Brainhack events, and https://www.brainhack.org [1] for a full list of projects.

### Event organization

Brainhack events span 1 to 4 days and include a variety of content to make them both accessible and fruitful for a wide range of attendees. The format of the events is not fixed, but varies based on the needs determined by the local organizing committee. At past events, the schedule has included various components aiming to quickly integrate the attendees and generate an environment conducive to productive collaboration. We have found the activity categories included in Table 2 to be valuable elements of creating a productive environment.

**Table 2:** Programming components of Brainhack events

∙**Meet and greet:** Brainhack events begin with a welcome to the hosting facility by the local organizing committee, along with a briefing about the event schedule, procedures, or other information that might be important for the attendees.
∙**Ice breaker:** Interaction between attendees is the key to a successful Brainhack event. The ice breaker is an activity to introduce attendees and their interests to one another. One strategy that has been successful is for each attendee to give their name, their institution, and three words that describe their interests. Such ice breakers could take various forms, including a speed-dating paradigm in which attendees pair up for a brief conversation, after which attendees swap partners, and this continues until every pair has met.
∙**Ignite talks:** Brainhack’s equivalent of keynote sessions, Ignite talks are inspirational talks on the big picture of open brain science that are intended to invigorate the audience for the day ahead. These are ideally brief (a 10-minute presentation followed by 10 minutes of questions), of general relevance, and are provided by a luminary in the field.
∙**Hacking:** The core of Brainhack is ‘open hacking’ sessions during which attendees collaborate together on projects of their choosing. Attendees who have specific project ideas or data that they would like to explore are encouraged to advertise their project at https://www.brainhack.org [1] prior to the event. At the opening of the Brainhack event, typically after the ice breaker, attendees pitch their ideas and afterwards mingle with others to organize a project team. Teams work together throughout the remainder of Brainhack and are given the opportunity to present their progress during the wrap-up session at the end of the event.
∙**Brainhacking 101:** The educational track enables less experienced attendees to learn basic software and data analysis skills. Occurring in parallel so as not to interfere with the ongoing hacking sessions, this track begins with ‘Installfest’ sessions during which attendees receive help installing any required software. Afterwards there are several hands-on tutorials that cover topics like: using Github, Python programming, using Python to load and visualize neuroimaging data, and performing meta-analyses of scientific literature. The resources for educational sessions are made freely available online (e.g., [31]).
∙**Unconference:** Sessions for attendees to present their research or other topics of current interest. Immediately prior to these sessions, the agenda is determined on-site. Attendees who are willing to present add their name to a sign-up sheet and in the event that there are more interested presenters than time, the group is polled to determine which presentations are given time or to extend the amount of time allotted. Instead of unconference sessions, some sites have incorporated ‘data blitzes,’ consisting of a pre-organized session where attendees have the opportunity to present their research. Brainhack Miami has had success with this model and has secured funding to award monetary prizes to the best presentations.
∙**Wrap-up and feedback:** Brainhack events typically finish with a wrap-up session during which project teams describe the progress that they made or give a demo of their results in a brief (∼1–2-minute) presentation. Afterwards, the local organizers lead a discussion about what worked well with the event, and how it could be improved in the future.

#### Practical considerations

To enable broad attendance by researchers, regardless of their resources, fees are kept as low as possible and many of the events are free. This has been made possible through generous financial support and meeting space provided by hosting institutions and corporate sponsors. When possible, lunch and dinner have been provided to encourage continued interaction between attendees throughout the duration of the event. We have also aimed to reduce travel costs by co-locating events with other international workshops, such as the 2012 Biennial Conference on Resting State Brain Connectivity in Magdeburg, Germany, and annual meetings of the Organization of Human Brain Mapping (OHBM).

#### Partnership with OHBM

The first annual OHBM Hackathon took place in Seattle, Washington as an initiative of the meeting’s 2013 local organizing committee, and its format has evolved subsequently to complement and bring value to the mainstream meeting. The 2013 event followed a traditional model of competitive challenges to accelerate the adoption of cloud-based open neuroscience tools by the neuroimaging community, while introducing a novel, conference-long ‘Data Science Room’ for collaboration. In 2014, Brainhack’s collaborative ethos was integrated, eliciting a strong, positive response from participants, and the OHBM leadership has continued to be very supportive of the Brainhack model ever since. The annual OHBM Hackathon has evolved to include a 2-day intensive hackathon and an additional educational day that is open to all OHBM attendees, and continues to include the Data Science Room to host educational content and open collaboration throughout the meeting.

#### Regional events

Brainhack began as international events that drew attendees from all over the world to work together in open collaboration. Early interest in transferring this model to the local level was tempered by fears that local events would not be able to provide enough content to attract attendees. Brainhack Eastern Daylight Time (Brainhack EDT) was developed to address these concerns by organizing several simultaneous events that are virtually linked to enable the real-time sharing of content across sites. Events were limited to sites in time zones within 1 hour of EDT to simplify scheduling. This innovative distributed model drew 242 attendees across seven sites located in three different countries, and has since been followed by Brainhack Americas, which extended this model to the entirety of North, South, and Central America (see Tables 3 and 4).

**Table 3:** Brainhack events occurring 2012–2014

**2012 Brainhack and Unconference**				1–4 September 2012
	Leipzig, Germany (72)			
		Host: Max Planck Institute for Human Cognition and Brain Sciences		
		Organizers: Daniel Margulies ^⋆^, Pierre Bellec, Cameron Craddock, Donald McLaren,		
		Maarten Mennes		
**OHBM Hackathon 2013**				16–20 June 2013
	Seattle, Washington, USA (136)			
		Host: Organization for Human Brain Mapping		
		Organizers: Nolan Nichols ^⋆^, Tom Grabowski ^⋆^, Chinh Dang, Elaine Shen,		
		Rachel Pizarro, Jamie Kinney, Satra Ghosh, and the OHBM 2013 Local Organizing Committee		
**Brainhack 2013**				23–26 October 2013
	Paris, France (70)			
		Host: Laboratoire d’Imagerie Biomédicale, Sorbonne Universités, Université Pierre-et-		
		Marie-Curie, Paris 06, CNRS, INSERM		
		Organizers: Selma Mesmoudi ^⋆^, Yves Burnod ^⋆^, Donald McLaren, Cameron Craddock,		
		Pierre Bellec, Daniel Margulies, Maarten Mennes		
**OHBM Hackathon 2014**				5–7 July 2014
	Berlin, Germany (65)			
		Host: Organization for Human Brain Mapping		
		Organizers: Daniel Margulies ^⋆^, Pierre Bellec, Cameron Craddock, Tom Grabowski,		
		Sean Hill, Nolan Nichols, JB Poline, and the OHBM 2014 Local Organizing Committee		
**Brainhack Eastern Daylight Time**				18–19 October 2014
	Ann Arbor, Michigan, USA (15)			
		Host: University of Michigan		
		Organizers: Scott Peltier ^⋆^, Robert Welsh ^⋆^		
	Boston, Massachusetts, USA (35)			
		Host: Massachusetts Institute of Technology		
		Organizers: Satra Ghosh ^⋆^, Matt Hutchison ^⋆^, Donald McLaren ^⋆^		
	Miami, Florida, USA (39)			
		Host: Florida International University		
		Organizers: Angie Laird ^⋆^, Lucina Uddin ^⋆^		
	Montréal, Québec, Canada (49)			
		Host: Centre de recherche de l’Institut universitaire de gériatrie de Montréal		
		Organizers: Benjamin De Leener ^⋆^, Julien Cohen-Adad ^⋆^, Pierre Bellec ^⋆^		
	New York, New York, USA (37)			
		Host: Child Mind Institute and Columbia University		
		Organizers: Cameron Craddock ^⋆*‡*^, Andrew Gerber ^⋆^		
	Porto Alegre, Brazil (25)			
		Host: Pontifícia Universidade Católica do Rio Grande do Sul		
		Organizers: Alexandre Franco ^⋆^, Caroline Fröhlich ^⋆^, Felipe Meneguzzi ^⋆^		
	Toronto, Ontario, Canada (15)			
		Host: University of Toronto		
		Organizers: Jonathan Downar ^⋆^, Katie Dunlop ^⋆^, Stephen Strother ^⋆^		
	Washington DC, USA (27)			
		Host: Georgetown University		
		Organizers: John Van Meter ^⋆^, Lei Liew ^⋆^, Ziad Saad ^⋆^, Prantik Kundu ^⋆^		

The number of attendees for each event are included in parentheses ^⋆^Local organizers for an event. ^*‡*^Distributed event main organizer

**Table 4:** Brainhack events in 2015

**OHBM Hackathon 2015**				12–14 June 2015
	Honolulu, Hawaii, USA (59)			
		Host: Organization for Human Brain Mapping		
		Organizers: Nolan Nichols ^⋆^, Pierre Bellec, Cameron Craddock, Tom Grabowski,		
		Jack Van Horn, Daniel Margulies, JB Poline, and the OHBM 2015 Local Organizing Committee		
**Brainhack Montreal 2015**				27–29 July 2015
	Montréal, Québec, Canada (53)			
		Host: Centre de recherche de l’Institut universitaire de gériatrie de Montréal		
		Organizers: Benjamin De Leener ^⋆^, Julien Cohen-Adad ^⋆^, Pierre Bellec ^⋆^		
		Sebastien Dery ^⋆^, Pierre-Olivier Quirion ^⋆^		
**Brainhack Americas**				23–25 October 2015
	Ann Arbor, Michigan, USA (10)			
		Host: University of Michigan		
		Organizers: Scott Peltier ^⋆^, Robert Welsh ^⋆^		
	Berkeley, California, USA (7)			
		Host: D-Lab, University of California, Berkeley		
		Organizers: Daniel Lurie ^⋆^, JB Poline ^⋆^		
	Los Angeles, California, USA (38)			
		Host: University of Southern California		
		Organizers: Lei Liew ^⋆^, Gautam Prasad ^⋆^, Yonggang Shi ^⋆^		
	Miami, Florida, USA (50)			
		Host: University of Miami		
		Organizers: Lucina Uddin ^⋆^, Angie Laird ^⋆^		
	New York, New York, USA (33)			
		Host: Translational & Molecular Imaging Institute, Icahn School of Medicine		
		at Mount Sinai and Child Mind Institute		
		Organizers: Christopher Cannistraci ^⋆^, Prantik Kundu ^⋆^, David O’Connor,		
		Ting Xu, Cameron Craddock ^*‡*^,		
	Porto Alegre, Brazil (30)			
		Host: Pontifícia Universidade Católica do Rio Grande do Sul		
		Organizers: Alexandre Franco ^⋆^, Anibal Sólon Heinsfeld, Felipe Meneguzzi ^⋆^, Ramon Fraga Pereira		
	Querétaro, México (31)			
		Host: Instituto De Neurobiología, Universidad Nacional Autónoma de México		
		Organizers: Sarael Alcauter ^⋆^, Fernando Barrios ^⋆^, Cameron Craddock ^*‡*^,		
		Eric H Pasaye ^⋆^		
	Seattle, Washington, USA (12)			
		Host: University of Washington eScience Institute		
		Organizers: Ariel Rokem ^⋆^		

The number of attendees for each event are included in parentheses ^⋆^Local organizers for an event. ^*‡*^Distributed event main organizer

### Post-conference publications

While traditional conference publications are submitted in advance of the event, to complement the unique on-site organizational structure of Brainhack, a new publication paradigm is needed that accommodates its open format. In partnership with *GigaScience* [2] we have recently introduced project reports in the form of post-conference proceedings as a way to account for and promote the progress made at Brainhacks. Proceedings will be published annually, peer-reviewed by members of the Brainhack community, and are open to submissions from all the previous year’s Brainhack events. Further information can be found at https://www.brainhack.org/proceedings [3].

### Brainhack thematic series

Additionally, *GigaScience* [2] is hosting a Brainhack Thematic Series as a venue for publishing full research articles that feature open tools for neuroscience. The series invites submissions that embody the ethos of open science. Example topics include open source software projects, data repositories, meta-analytic and collaborative resources, and other open science initiatives, regardless of whether they have roots at Brainhack events. More information can be found at https://www.brainhack.org/series [4].

## Conclusions

Brainhack promotes open neuroscience by offering unique opportunities to researchers from a variety of backgrounds to build collaborations and develop new skills. It is particularly valuable to junior researchers and those from developing economies who have limited opportunities to interact with peers and senior scientists outside their home institutions. Despite these successes, Brainhack is a nascent concept for scientific meetings, and there remains substantial room for innovation. To this end, we are excited to announce the Brainhack Proceedings and Brainhack Thematic Series for providing researchers with tangible scientific credit for their contributions to Brainhack events and open science. For the future, we will be working to expand the global audience of Brainhack by hosting events throughout Asia.

## Availability of supporting data

More information about Brainhack, including projects from past and future events, can be found at https://www.brainhack.org [1].

## References

[1] Brainhack web page. http://www.brainhack.org. Accessed: 21 Dec 2015.

[2] GigaScience web page. http://www.gigasciencejournal.com/. Accessed: 21 Dec 2015.

[3] Brainhack Proceedings call for submissions. http://brainhack.org/proceedings. Accessed: 21 Dec 2015.

[4] Brainhack Thematic Series call for submissions. http://www.brainhack.org/series. Accessed: 21 Dec 2015.

[5] Inscapes video on Vimeo. http://vimeo.com/67962604. Accessed: 21 Dec 2015.

[6] Vanderwal T, Kelly C, Eilbott J, Mayes LC, Castellanos FX (2015). Inscapes: A movie paradigm to improve compliance in functional magnetic resonance imaging. NeuroImage.

[7] ABIDE Preprocessing Initiative web page. http://preprocessed-connectomes-project.github.io/abide. Accessed: 21 Dec 2015.

[8] ABIDE Initiative web page. http://fcon_1000.projects.nitrc.org/indi/abide. Accessed: 21 Dec 2015.

[9] Di Martino A, Yan CG, Li Q, Denio E, Castellanos FX, Alaerts K, Anderson JS, Assaf M, Bookheimer SY, Dapretto M, Deen B, Delmonte S, Dinstein I, Ertl-Wagner B, Fair DA, Gallagher L, Kennedy DP, Keown CL, Keysers C, Lainhart JE, Lord C, Luna B, Menon V, Minshew NJ, Monk CS, Mueller S, Muller RA, Nebel MB, Nigg JT, O’Hearn K, Pelphrey KA, Peltier SJ, Rudie JD, Sunaert S, Thioux M, Tyszka JM, Uddin LQ, Verhoeven JS, Wenderoth N, Wiggins JL, Mostofsky SH, Milham MP (2014). The autism brain imaging data exchange: towards a large-scale evaluation of the intrinsic brain architecture in autism. Mol Psychiatry.

[10] Connectome Computation System web page. http://lfcd.psych.ac.cn/ccs.html. Accessed: 21 Dec 2015.

[11] Xu T, Yang Z, Jiang L, Xing XX, Zuo XN (2015). A connectome computation system for discovery science of brain. Sci Bull.

[12] Configurable Pipeline for the Analysis of Connectomes web page. http://fcp-indi.github.io. Accessed: 21 Dec 2015.

[13] Craddock C, Sikka S, Cheung B, Khanuja R, Ghosh SS, Yan C, Li Q, Lurie D, Vogelstein J, Burns R, Colcombe S, Mennes M, Kelly C, Di Martino A, Castellanos FX, Milham M. Towards automatanalysis of connectomes: The configurable pipeline for the analysis of connectomes (c-pac). Front Neuroinformatics. 42: doi:http://dx.doi.org/10.3389/conf.fninf.2013.09.00042.

[14] Data Preprocessing Assistant for Resting State fMRI web page. http://rfmri.org/DPARSF. Accessed: 21 Dec 2015.

[15] Chao-Gan Y, Yu-Feng Z (2010). DPARSF: A MATLAB Toolbox for “Pipeline” Data Analysis of Resting-State fMRI. Front Syst Neurosci.

[16] Neuro Imaging Analysis Kit web page. https://www.nitrc.org/projects/niak/. Accessed: 21 Dec 2015.

[17] Bellec P, Lavoie-Courchesne S, Dickinson P, Lerch JP, Zijdenbos AP, Evans AC. The pipeline system for Octave and Matlab (PSOM): a lightweight scripting framework and execution engine for scientific workflows. Front Neuroinformatics. 2012; 6:7. ISSN:1662-5196, doi:http://dx.doi.org/10.3389/fninf.2012.00007, http://www.frontiersin.org/neuroinformatics/10.3389/fninf.2012.00007/abstract. 10.3389/fninf.2012.00007 PMC3318188 22493575

[18] Freesurfer web page. http://freesurfer.net/. Accessed: 21 Dec 2015.

[19] Fischl B, Dale AM (2000). Measuring the thickness of the human cerebral cortex from magnetic resonance images. Proc Natl Acad Sci USA.

[20] CIVET web page. http://www.bic.mni.mcgill.ca/ServicesSoftware/CIVET. Accessed: 21 Dec 2015.

[21] Zijdenbos AP, Forghani R, Evans AC (2002). Automatic “pipeline” analysis of 3-D MRI data for clinical trials: application to multiple sclerosis. IEEE Trans Med Imaging.

[22] Advanced Normalization Tools web page. http://picsl.upenn.edu/software/ants/. Accessed: 21 Dec 2015.

[23] Tustison NJ, Cook PA, Klein A, Song G, Das SR, Duda JT, Kandel BM, van Strien N, Stone JR, Gee JC, Avants BB (2014). Large-scale evaluation of ANTs and FreeSurfer cortical thickness measurements. Neuroimage.

[24] Valk SL, Di Martino A, Milham MP, Bernhardt BC (2015). Multicenter mapping of structural network alterations in autism. Hum Brain Mapp.

[25] Mennes M, Jenkinson M, Valabregue R, Buitelaar JK, Beckmann C, Smith S (2014). Optimizing full-brain coverage in human brain MRI through population distributions of brain size. NeuroImage.

[26] Das S, Zijdenbos AP, Vins D, Harlap J, Evans AC. Loris: A web-based data management system for multi-center studies. Front Neuroinformatics. 2012;5(37): doi:http://dx.doi.org/10.3389/fninf.2011.00037. 10.3389/fninf.2011.00037 PMC3262165 22319489

[27] Daydreaming app web page. http://daydreaming-the-app.net. Accessed: 21 Dec 2015.

[28] Clubs of Sceince web page. http://dery.xyz/projects/templates/clubsofscience.html. Accessed: 21 Dec 2015.

[29] linkRbrain web page. http://www.linkrbrain.xyz/. Accessed: 21 Dec 2015.

[30] Mesmoudi S, Rodic M, Cioli C, Cointet JP, Yarkoni T, Burnod Y (2015). Linkrbrain: Multi-scale data integrator of the brain. J Neurosci Methods.

[31] Brainhacking 101 Github page. https://github.com/ohbm/brain-hacking-101. Accessed: 21 Dec 2015.

